# Simultaneous dengue and COVID-19 epidemics: Difficult days ahead?

**DOI:** 10.1371/journal.pntd.0008426

**Published:** 2020-08-14

**Authors:** Mathieu Nacher, Maylis Douine, Mélanie Gaillet, Claude Flamand, Dominique Rousset, Cyril Rousseau, Chedli Mahdaoui, Stanley Carroll, Audrey Valdes, Nathalie Passard, Gabriel Carles, Félix Djossou, Magalie Demar, Loïc Epelboin

**Affiliations:** 1 Centre d’Investigation Clinique Antilles Guyane, CIC INSERM 1424, Centre Hospitalier Andrée Rosemon, Cayenne, French Guiana; 2 DFR Santé, Université de Guyane, Cayenne, French Guiana; 3 Centres Délocalisés de Prévention et de Soins, Centre Hospitalier de Cayenne, Cayenne, French Guiana; 4 Unité d’épidémiologie, Institut Pasteur de la Guyane, Cayenne, French Guiana; 5 Centre National de Référence Arbovirus et virus respiratoires, Institut Pasteur de la Guyane, Cayenne, French Guiana; 6 Santé Publique France, CIRE Antilles Guyane, Cayenne, French Guiana; 7 Maison de Garde, Centre Hospitalier de Cayenne, Cayenne, French Guiana; 8 Réseau de médecins sentinelles, Cayenne, French Guiana; 9 Hygiène, Centre Hospitalier de Cayenne, Cayenne, French Guiana; 10 CPIAS, Centre Hospitalier de Cayenne, Cayenne, French Guiana; 11 Service d’obstétrique, centre hospitalier de l’Ouest Guyanais, French Guiana; 12 Service des Maladies Infectieuses et Tropicales, Centre Hospitalier de Cayenne, Cayenne, French Guiana; 13 Laboratoire, Centre Hospitalier de Cayenne, Cayenne, French Guiana; 14 TBIP, Université de Guyane, Cayenne, French Guiana; Faculty of Science, Ain Shams University (ASU), EGYPT

## Introduction

As the coronavirus disease 2019 (COVID-19) pandemic reaches South America, researchers have already reported concerns about the impact that significant co-circulation of the dengue viruses and COVID-19 could have on the health system [1–3]. In Singapore, 2 clinical cases consisted of dengue-like syndromes with thrombocytopenia and false immunoglobulin M (IgM) positives with 2 different serological kits in patients who were finally shown to have COVID-19 [4]. In addition to differential diagnosis, there is always the possibility of coinfections by both COVID-19 and dengue virus, as was recently described in Mayotte [5]. It is not yet known whether such coinfections will lead to greater severity, but it should be a point of vigilance. Coinfections with different pathogens may result in complex and unpredictable consequences on severity. The literature suggests that co-infections with flu and dengue may be associated with greater severity [6,7]. In French Guiana and the Amazonian region of Brazil, the coinfection between malaria and dengue is not exceptional [8,9]. Arboviral infections (Tonate, Mayaro) [10], Q fever [11], leptospirosis, influenza, Amazonian toxoplasmosis, and primary HIV infection can be potential differential diagnoses during a dengue epidemic [12]. The first 24 to 48 hours are important due to similar signs before the clinical differentiation of the different diseases.

**Fig 1:**
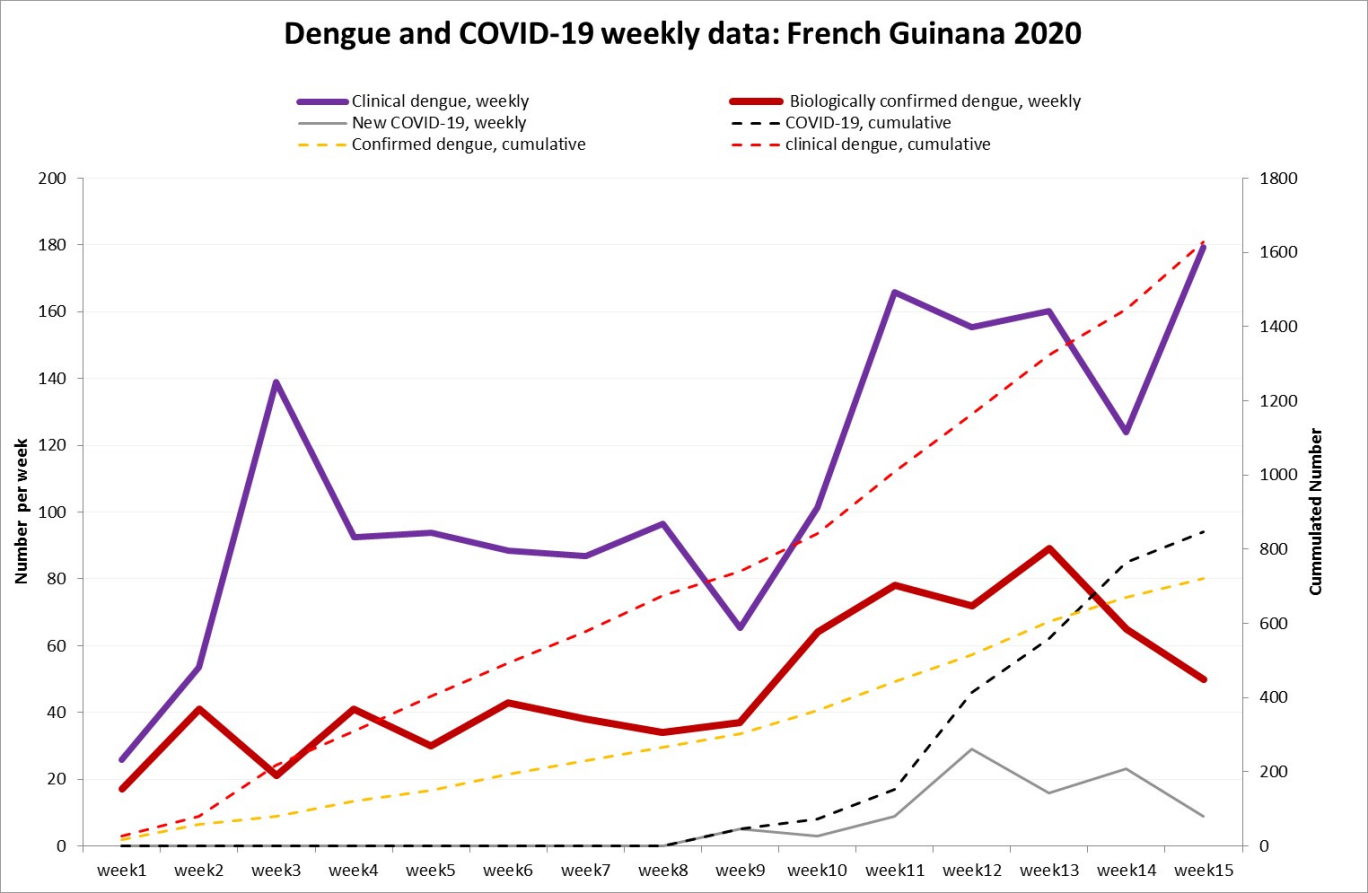
Dengue and COVID-19 data: French Guiana, January–April 2020. COVID-19, coronavirus disease 2019.

In April 2020, 7 years after the last epidemic, the Committee for Infectious and Emergent Diseases officially declared a dengue epidemic (dengue virus 1 [DENV-1] and 2) on the Maroni River, in the Kourou area, and in the Cayenne Area [13]. This occurs simultaneously with the COVID-19 pandemic (Fig 1), and the perspective of joint epidemics has several complex collateral consequences.

## Is it COVID-19 or dengue?

Contextual elements are surely important to orient clinicians. While some clinical signs may point to COVID-19 or dengue in case series, at the individual patient scale, the imperfect positive predictive value and clinical variability do not guarantee a diagnosis of certainty (Table 1). Thus, some studies report 25% of patients with confirmed dengue having a cough and 20% with upper respiratory tract symptoms [14]. Similarly, COVID-19 may manifest itself as fever with muscle and joint pains without respiratory symptoms, especially in infants [15–17]. Thus, most patients must be explored for both diseases.

**Table 1:** COVID-19 and dengue fever similarities and differences.

	COVID-19	Dengue
**Symptoms and biological findings**		
Fever	+++	+++
Headache	++	+++
Retro-orbital pain^a^		++
Asthenia	+	++
Rash	+	++
Purpura^a^		++
Myalgia/arthralgia	+	++
Dyspnea^b^	++	
Anorexia	+	+
Cough^b^	+++	+
Chest pain^b^	++	
Cyanosis^b^	+	
Pharyngitis^b^	++	++
Rhinorrhea^b^	+	
Sneezing^b^	+	
Anosmia, ageusia^b^	+++	± (dysgueusia)
Diarrhea	+	+
Nausea/vomiting	+	+
Persistent vomiting^a^		+*
Abdominal pain^a^		++*
Consciousness alteration	+	+*
Agitation	+*	+*
**Biology**		
CRP^b^	++*	
Procalcitonin^b^	+*	
Leukocytes^b^		↓
Neutrophils^b^	Relative ↑	Relative ↓
Lymphocytes^b^	Relative ↓	Relative ↓
Platelets	↓*	↓
Ferritin	↑*	↑*
Hemoglobin		↑*
Serum protein/albumin	↓*	↓*
O_2_ saturation	↓*	↓*
PO_2_	↓*	↓*
ASAT/ALAT	↑	↑
LDH	↑*	↑*
D-dimers^b^	↑*	
Troponin^b^	↑*^#^	
Sodium	↓*	↓*
Potassium	↓*	
Calcium	↓*	

^a^Indicates more discriminating criteria DENGUE.

^b^Indicates more discriminating criteria COVID-19.

*Prognostic value.

^#^The American College of Cardiology recommends not to prescribe it unless suspected of infarction.

**Abbreviations:** ALAT, alanine amino transferase; ASAT, aspartate amino transferase; COVID-19, coronavirus disease 2019; CRP, C-reactive protein; LDH, lactate dehydrogenase

However, given the safety constraints linked to COVID-19, any nonurgent febrile patient must be seen and tested in a special sector where great precautions are taken to avoid the risk of transmission [18]. This creates organizational bottlenecks. Because of this great disruption in the organization of care, until recently it was only after receiving the results (24–48 hours for negative results) that further explorations were performed, which for a while led to potentially dangerous diagnostic delays in patients with dengue. Indeed, the critical period is between 3 and 8 days, usually at the time of defervescence, and a 48-hour delay in this context can be a loss of chance for patients with dengue, as this disease may be potentially lethal as well [19,20]. For nonfebrile patients with rhinopharyngitis and cough, dengue may not be a first-line diagnosis.

In the context of cocirculation of 2 potentially fatal viruses, it is key to enable a combined diagnosis (COVID-19–dengue) for ambulatory patients and at least complete blood count, liver enzymes, C-reactive protein, serum protein, creatinine, and electrolytes. For patients who require hospitalization, the logistical problem is less complicated, and all the necessary samples are taken. In a context where hospital beds are sparse and patients are mostly confined at home with telemedicine follow-up, dyspnea, cyanosis, impaired consciousness, fainting, shock, hemorrhagic signs, and jaundice—initially or during follow-up—should be points of vigilance in order to hospitalize patients if needed.

## Telemedicine or normal consultation?

Given the potential risks of infectious diseases in pregnancy, and the difficulties to evaluate small children, infants and pregnant women should preferably have normal consultations. Patients with signs of severity requiring hospitalization are treated in the hospital with the necessary precautions until the diagnosis is made. For other patients, they can consult the hospital in a specific location for diagnosis and follow-up of fever during COVID-19 + dengue epidemics. For patients using teleconsultations, paraclinical tests should be performed at one of the COVID-19 + dengue diagnostic centers [21]. Follow-up can be monitored by regular teleconsultations, with the possibility, if necessary, of home visits by nurses or a pool of doctors equipped with personal protective equipment (PPE) to check the constants and collect samples.

## Other differential diagnoses?

The current differential diagnoses in French Guiana are as follows: In a context of respiratory signs and symptoms, Q fever, usual respiratory pathogens such as *Streptococcus pneumoniae* and *Mycoplasma pneumoniae*, influenza, leptospirosis, and Amazonian toxoplasmosis; dengue-like clinical presentations may also be caused by other arboviruses such as Tonate virus and Mayaro virus (French Guiana reports no case of chikungunya virus infection from early 2016 and no case of Zika virus infection from early 2017) [22], malaria, Q fever, leptospirosis, salmonellosis, and HIV primary infection [10,12,23,24]. This list will vary between regions. The objectives of paraclinical explorations are 2-fold: to make the differential diagnosis and to look for signs of severity (white blood cell and platelet counts, C-reactive protein, serum electrolytes, aspartate amino transferase, alanine amino transferase, bilirubin, prothrombin time, activated thromboplastin time, and lactate dehydrogenase [LDH]). According to the context and clinical presentation, physicians may prescribe a malaria test, blood and urine cultures, serologies, or molecular diagnosis of differential diagnoses.

## What is the impact of social distancing on dengue epidemics?

In French Guiana, with social distancing, vector control has been scaled back by only relying on vehicle spraying of neighborhoods with deltamethrin to attempt—in a context of widespread insecticide resistance—to reduce vector numbers. However, intra-domiciliary or compound interventions to destroy larvae or inspect for breeding places, or in-house spraying, have been interrupted. Maintenance of public spaces and gardens and collection of potential water receptacles have been drastically reduced. All this arguably has the potential to give an edge to the dengue fever vectors, *Aedes aegypti*. Population movements and the frequency of interactions are major drivers of epidemics [25]. Social distancing, while considered to double intra-domiciliary COVID-19 transmission, greatly reduces transmission at the population level [26]. There is also a close link between travel and dengue epidemics [27]. In French Guiana, daily airline connections between Guadeloupe, Martinique, and French Guiana are historical drivers of dengue virus circulation [28]. Currently, in French Guiana transmission has increased during social distancing, which was implemented simultaneously with the second rainy season. It is therefore difficult to disentangle the respective contributions of rainfall and suboptimal mosquito control measures due to social distancing. Transmission depends on the frequency of mosquito bites, and infection incidence in the human and mosquito populations is almost independent of the duration of contacts [27]. Mosquitoes are locally mobile but only travel short distances, usually far less than infected humans who can extend the epidemic potential. Modeling studies on the relation between population movements and dengue suggest that increased population movements should indeed increase the risk of epidemic [29,30]. If the mosquito population was not uniformly distributed, the transmission potential of dengue at the metapopulation level would be determined by the size of the largest subpopulation and would be reduced by stronger human-mediated connectivity between vector populations. The extinction of the dengue virus epidemic is less likely when increased human movement enhances the rescue effect. Thus, modeling suggests that infection hubs and reservoirs can be locations people visit frequently, but briefly, and the relative importance of human and mosquito populations in maintaining dengue depends on the distribution of the vector population and the variability in human travel patterns.

## Does COVID-19 social distancing have the potential to increase dengue morbidity?

With the lockdown, people stay home, and the risk of dengue infection may increase as *A*. *aegypti*, the vector of dengue virus, lays its eggs on the walls of water-filled containers in the house and its surroundings. Moreover, many persons are afraid to go to the hospital or to consult health professionals because they fear that other patients or health professionals have COVID-19. In French Guiana, despite a limited number of cases (97 on April 21, 2020), the number of consultations has greatly declined in part because nonurgent health problems are postponed but perhaps because of this fear of contamination. Moreover, given the safety constraints linked to COVID-19, any nonurgent febrile patient must be seen and tested in a special sector where great precautions are taken to avoid the risk of transmission. Because of this great disruption in the organization of care, until recently it was only after receiving the results (24–48 hours for negative results) that further explorations were performed, which for a while led to potentially dangerous diagnostic delays in patients with dengue.

## PPE and reagents

Although there have already been organizational complications capable of affecting the quality of care and vector control, due to the co-circulation of viruses there are additional aspects that are worrisome. In a context of global depletion of PPE, the increase of dengue circulation with around 150 (for the moment) suspected dengue patients per week (Fig 1) in a context of COVID-19 circulation will lead to a significant increase in PPE use because, with COVID-19 circulation, febrile patients require special protection until COVID-19 has been ruled out. This will further aggravate pressure on stocks that are already insufficient and challenging to replenish. Furthermore, the COVID-19 epidemic has led to global shortages in certain laboratory reagents. Hence, molecular diagnosis of COVID-19 and dengue requires extraction kits and enzymes that are the same and thus accelerate the depletion of stocks with uncertainties as to when it will be possible to restock. Thus, to spare reagents, nonstructural antigen 1 (NS-1) rapid tests are used despite their imperfect sensitivity, assuming their positive predictive value is good during an epidemic. Only negative NS-1 results are checked with real-time PCR and serology.

Another tension for the system is that health workers may be incapacitated by either disease. For COVID-19, it has been shown in China, Italy, and the US that a substantial proportion of health workers were infected by COVID-19, presumably more in emergency services [31,32]. In Mayotte, a French Island in the southwestern Indian Ocean, almost 20% of the more than 200 patients infected with COVID-19 are health workers. In French Guiana, several emergency physicians have already been infected [33]. Moreover, given the rapid turnover of health professionals rotating from mainland France, a large proportion of the staff is not immune to dengue, and past epidemics have often affected a significant proportion of the hospital workforce, another potential source of tension.

## What can we do now?

The population should be well aware of both dengue and COVID-19 and should be informed about prevention measures. Regarding vector control, health promotion should encourage populations to look out for potential vector breeding places and protect themselves from mosquito bites. Local authorities should be very vigilant and activate strategic services essential to vector control (waste management, maintenance of public spaces, intra-domiciliary interventions, notably around cases). Regarding COVID-19, testing of suspected cases should expand (it has been hampered by logistical constraints and insufficient supplies of swabs and reagents), and aggressive contact tracing and isolation should continue. Diagnosis of febrile patients should be organized to allow diagnosing of both dengue and COVID-19 without delays due to COVID-19 constraints. Hospitals, which have been radically reorganized to accommodate a surge of COVID-19 patients, should plan for severe dengue beds. Patients should remain under mosquito nets, to avoid infecting other mosquitoes. Finally, although during a single epidemic specific syndromes have good positive predictive value and do not always require biological confirmation, in this case, because of the co-circulation and the complicated collateral consequences, diagnostic confirmation seems mandatory throughout the epidemic, which will use more resources.

## What will happen in the months ahead?

The last dengue epidemic lasted a year (2012 week 40 to 2013 week 42) and 5 months with 500–700 weekly clinical cases reported by the sentinel network for a population of 260,000 [34]. The impact of social distancing relaxation on population movements and the epidemic is hard to predict, but it could further accelerate the circulation of the virus in mosquito populations. For COVID-19, for the moment the surge has not happened due to the early implementation of social distancing, border closure, and cancelation of most air traffic. Most initial cases were imported with secondary transmission clusters around imported cases. However, as social distancing relaxes in the weeks ahead and flights from France and French Caribbean (Martinique and Guadeloupe) resume, the potential for reigniting the epidemic will increase. Authorities will need to be very aggressive on contact tracing and vigilant on testing and tracking arriving passengers. With a single international airport this seems very feasible. However, a more difficult task is to control borders with Suriname and especially with Brazil. These borders largely consist of Amazonian forest and are difficult to control, as shown by the massive influx of illegal goldminers from Brazil in French Guiana. As the Brazilian president resists social distancing, there are risks of importing cases through the interior villages, a concern that also affects other neighboring countries. Furthermore, the state of Amapá, neighboring French Guiana, seems to be currently one of the most impacted in Brazil in terms of the number of COVID-19 patients in proportion to its population. The incidence on May 21 was 613.4 per 100,000 inhabitants, and fatalities were 17.9 per 100,000 inhabitants [35]. Several large clusters have appeared on the border with Brazil, which has now become the most active site for COVID-19. Suriname, a country bordering French Guiana, has so far registered 565 suspected cases, 11 people confirmed positive for COVID-19, and 1 death, according to the results communicated by the Surinamese authorities in May [36]. In this context, a simultaneous dengue epidemic will be a major problem for the healthcare systems in the region, and beyond.
